# Detection of left ventricular wall motion abnormalities from volume rendering of 4DCT cardiac angiograms using deep learning

**DOI:** 10.3389/fcvm.2022.919751

**Published:** 2022-07-28

**Authors:** Zhennong Chen, Francisco Contijoch, Gabrielle M. Colvert, Ashish Manohar, Andrew M. Kahn, Hari K. Narayan, Elliot McVeigh

**Affiliations:** ^1^Department of Bioengineering, UC San Diego School of Engineering, La Jolla, CA, United States; ^2^Department of Radiology, UC San Diego School of Medicine, La Jolla, CA, United States; ^3^Department of Mechanical and Aerospace Engineering, UC San Diego School of Engineering, La Jolla, CA, United States; ^4^Department of Cardiology, UC San Diego School of Medicine, La Jolla, CA, United States; ^5^Department of Pediatrics, UC San Diego School of Medicine, La Jolla, CA, United States

**Keywords:** computed tomography, left ventricle (LV), wall motion abnormality detection, volume rendering (VR), deep learning

## Abstract

**Background:**

The presence of left ventricular (LV) wall motion abnormalities (WMA) is an independent indicator of adverse cardiovascular events in patients with cardiovascular diseases. We develop and evaluate the ability to detect cardiac wall motion abnormalities (WMA) from dynamic volume renderings (VR) of clinical 4D computed tomography (CT) angiograms using a deep learning (DL) framework.

**Methods:**

Three hundred forty-three ECG-gated cardiac 4DCT studies (age: 61 ± 15, 60.1% male) were retrospectively evaluated. Volume-rendering videos of the LV blood pool were generated from 6 different perspectives (i.e., six views corresponding to every 60-degree rotation around the LV long axis); resulting in 2058 unique videos. Ground-truth WMA classification for each video was performed by evaluating the extent of impaired regional shortening visible (measured in the original 4DCT data). DL classification of each video for the presence of WMA was performed by first extracting image features frame-by-frame using a pre-trained Inception network and then evaluating the set of features using a long short-term memory network. Data were split into 60% for 5-fold cross-validation and 40% for testing.

**Results:**

Volume rendering videos represent ~800-fold data compression of the 4DCT volumes. Per-video DL classification performance was high for both cross-validation (accuracy = 93.1%, sensitivity = 90.0% and specificity = 95.1%, κ: 0.86) and testing (90.9, 90.2, and 91.4% respectively, κ: 0.81). Per-study performance was also high (cross-validation: 93.7, 93.5, 93.8%, κ: 0.87; testing: 93.5, 91.9, 94.7%, κ: 0.87). By re-binning per-video results into the 6 regional views of the LV we showed DL was accurate (mean accuracy = 93.1 and 90.9% for cross-validation and testing cohort, respectively) for every region. DL classification strongly agreed (accuracy = 91.0%, κ: 0.81) with expert visual assessment.

**Conclusions:**

Dynamic volume rendering of the LV blood pool combined with DL classification can accurately detect regional WMA from cardiac CT.

## Introduction

Left Ventricular (LV) wall motion abnormalities (WMA) are an independent indicator of adverse cardiovascular events and death in patients with cardiovascular diseases such as myocardial infarction (MI), dyssynchrony and congenital heart disease (1, 2). Further, regional WMA have greater prognostic values after acute MI than LV ejection fraction (EF) (3, 4). Multidetector computed tomography (CT) is routinely used to evaluate coronary arteries (5, 6). Recently, ECG-gated acquisition of cardiac 4DCT enables the combined assessment of coronary anatomy and LV function (7, 8). Recent publications show that regional WMA detection with CT agrees with echocardiography (9, 10) as well as with cardiac magnetic resonance (11, 12).

Dynamic information of the 3D cardiac motion and regional WMA is encoded in 4DCT data. Visualization of regional WMA with CT usually requires reformatting the acquired 3D data along standard 2D short- and long-axis imaging planes. However, it requires experience in practice to resolve the precise region of 3D wall motion abnormalities from these 2D planes. Further, these 2D plane views may be confounded by through-plane motion and foreshortening artifacts (13). We propose to directly view 3D regions of wall motion abnormalities through the use of volumetric visualization techniques such as volume rendering (VR) (14), which can preserve high resolution anatomical information and visualize 3D (15, 16) and 4D (17) data simultaneously over large regions of the LV in cardiovascular CT. In VR, the 3D CT volume is projected onto a 2D viewing plane and different colors and opacities are assigned to each voxel based on intensity. It has been shown that VR provides a highly representative and memory efficient way to depict 3D tissue structures and anatomic abnormalities (18, 19). In this paper, we perform dynamic 4D volume rendering by sequentially combining the VR of each CT time frame into a video of LV function (we call this video a “Volume Rendering video”). We propose to use volume rendering videos of 4DCT data to depict 3D motion dynamics and visualize highly local wall motion dynamics to detect regional WMA.

Analytical approaches to quantify 3D motion from 4DCT using image registration and deformable LV models have been developed (9, 20, 21). However, these approaches usually require complex and time-consuming steps such as user-guided image segmentation and point-to-point registration or feature tracking. Further, analysis of multiple frames at the native image resolution/size of 4DCT can lead to significant memory limitations (22), especially when running deep learning experiments using current graphical processing units (GPU). Volume rendering (VR) videos provide a high-resolution representation of 4DCT data which clearly depicts cardiac motion at a significantly reduced memory footprint (~1 Gigabyte when using original 4DCT for motion analysis and only 100 kilobytes when using volume rendering video). Given the lack of methods currently available to analyze motion observed in VR videos, we sought to create an objective observer that could automate VR video interpretation. Doing so would facilitate clinical adoption as it would avoid the need for training individuals on VR video interpretation and the approach could be readily shared. Deep learning approaches have been successfully used to perform classification of patients using medical images (23, 24). Further, DL methods, once trained, are very inexpensive and can be easily deployed.

Therefore, in this paper, we propose a novel framework which combines volume rendering videos of clinical cardiac CT cases with a DL classification to detect WMA. We outline a straightforward process to generate VR videos from 4DCT data and then utilize a combination of a convolutional neural network (CNN) and recurrent neural network (RNN) to assess regional WMA observable in the videos.

## Methods and materials

### CT data collection

Under institutional review board approval, 343 ECG-gated contrast enhanced cardiac CT patient studies between Jan 2018 and Dec 2020 were retrospectively collected with waiver of informed consent. Inclusion criteria were: each study (a) had images reconstructed across the entire cardiac cycle, (b) had a field-of-view which captured the entire LV, (c) was free from significant pacing lead artifact in the LV and (d) had a radiology report including assessment of cardiac function. Images were collected by a single, wide detector CT scanner with 256 detector rows (Revolution scanner, GE Healthcare, Chicago IL) allowing for a single heartbeat axial 16cm acquisition across the cardiac cycle. The CT studies were performed for range of clinical cardiac indications including suspected coronary artery disease (CAD, *n* = 153), pre-procedure assessment of pulmonary vein isolation (PVI, *n* = 126), preoperative assessment of transcatheter aortic valve replacement (TAVR, *n* = 42), preoperative assessment of left ventricular assist device placement (LVAD, *n* = 22).

### Production of volume rendering video of LV blood-pool

**Figure 1:**
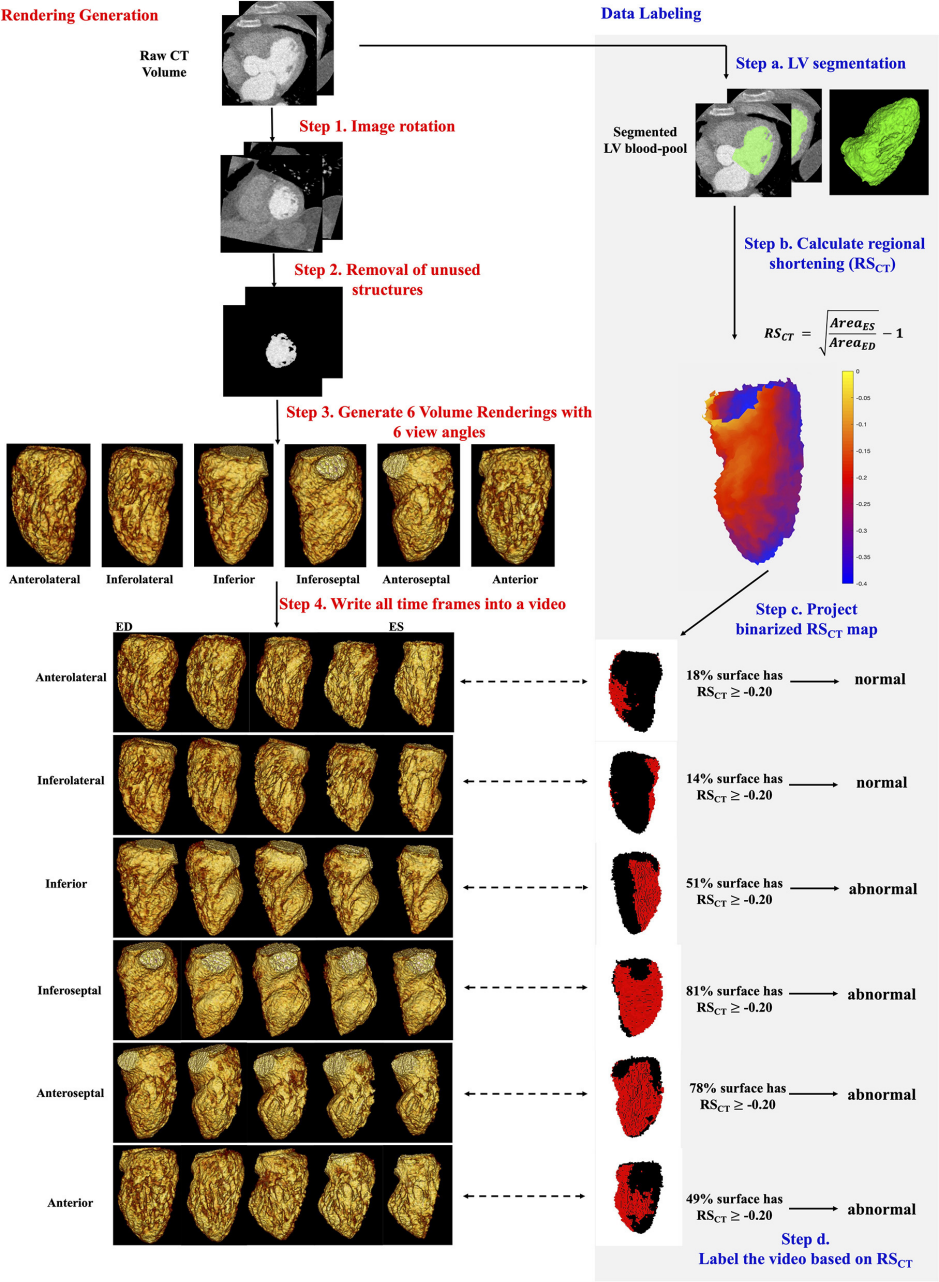
Automatic generation and quantitative labeling of volume rendering video. This figure contains two parts: Rendering Generation: automatic generation of VR video (left column, white background, step 1-4 in red) and Data Labeling: quantitative labeling of the video (right column, light gray background, step a-d in blue). Rendering Generation: *Step 1 and 2*: Prepare the greyscale image of LV blood-pool with all other structures removed. *Step 3*: For each study, 6 volume renderings with 6 view angles rotated every 60 degrees around the long axis were generated. The mid-cavity AHA segment in the foreground was noted under each view. *Step 4*: For each view angle, a volume rendering video was created to show the wall motion across one heartbeat. Five systolic frames in VR video were presented. ED, end-diastole; ES, end-systole. Data Labeling: *Step* a: LV segmentation. LV, green. *Step b*: Quantitative RS_CT_ was calculated for each voxel. *Step c*: The voxel-wise RS_CT_ map was binarized and projected onto the pixels in the VR video. See Supplementary Material 2 for more details. In rendered RS_CT_ map, the pixels with RS_CT_ ≥−0.20 (abnormal wall motion) were labeled as red and those with RS_CT_ < −0.20 (normal) were labeled as black. *Step d*: a video was labeled as abnormal if >35% endocardial surface has RS_CT_ ≥−0.20 (red pixels).

Figure 1 step 1-4 shows the pipeline of VR video production. The CT images were first rotated using visual landmarks such as the RV insertion and LV apex, so that every study had the same orientation (with the LV long axis along the z-axis of the images and the LV anterior wall at 12 o'clock in cross-sectional planes). Structures other than LV blood-pool (such as LV myocardium, ribs, the right ventricle, and great vessels) were automatically removed by a pre-trained DL segmentation U-Net (25) which has previously shown high accuracy in localizing the LV in CT images (25, 26). If present, pacing leads were removed manually.

The resultant grayscale images of the LV blood-pool (as shown in Fig. 1 step 2) were then used to produce Volume renderings (VR) *via* MATLAB (version: 2019b, MathWorks, Natick MA). Note the rendering was performed using the native CT scan resolution. The LV endocardial surface shown in VR was defined by automatically setting the intensity window level (WL) equal to the mean voxel intensity in a small ROI placed at the centroid of the LV blood pool and setting the window width (WW) equal to 150 HU (thus WL is study-specific, and WW is uniform for every study). Additional rendering parameters are listed in Supplementary Materials 1A. VR of all frames spanning one cardiac cycle was then saved as a video (“VR video,” Figure 1).

Each VR video projects the 3D LV volume from one specific projection view angle θ, thus it shows only part of the LV blood-pool and misses parts that are on the backside. Therefore, to see and evaluate all AHA segments, 6 VR videos were generated per study, with six different projection views θ_60×*n, n*∈[0, 1, 2, 3, 4, 5]_ corresponding to 60-degree rotations around the LV long axis (Supplementary Materials 1B for details**)**. With our design, each projection view had a particular mid-cavity AHA segment shown on the foreground (meaning this segment was the nearest to and in front of the ray source-point of rendering) as well as its corresponding basal and apical segments. Two adjacent mid-cavity AHA segments and their corresponding basal and apical segments were shown on the left and right boundary of the rendering in that view. In standard regional terminology, the six projection views (*n* = 0, 1, 2, 3, 4, 5 in θ_60×*n*_) looked at the LV from the view with mid-cavity Anterolateral, Inferolateral, Inferior, Inferoseptal, Anteroseptal and Anterior segments on the foreground, respectively. In this paper, to simplify the text we call them six “regional LV views” from anterolateral to anterior. In total, a *large* dataset of 2058 VR videos (343 patients × 6 views) with unique projections were generated.

### Classification of wall motion

Figure 1 steps a-d shows how the ground truth presence or absence of WMA at each location on the endocardium was determined. It is worth clarifying first that the ground truth is made on the original CT data not the volume rendered data. First, voxel-wise LV segmentations obtained using the U-Net were manually refined in ITK-SNAP (Philadelphia, PA, USA) (27). Then, regional shortening (RS_CT_) (8, 28, 29) of the endocardium was measured using a previously-validated surface feature tracking (21) technique. The accuracy of RS_CT_ in detecting WMA has been validated previously with strain measured by tagged MRI (12) [a validated non-invasive approach for detecting wall motion abnormalities in myocardial ischemia (30, 31)]. Regional shortening was calculated at each face on the endocardial mesh as:


$$\begin{array}{l} RS_{CT}=\sqrt{\frac{Area_{ES}}{Area_{ED}}}-1 \end{array}$$

where Area_ES_ is the area of a local surface mesh at end-systole (ES) and Area_ED_ is the area of the same mesh at end-diastole (ED). ED and ES were determined based on the largest and smallest segmented LV blood-pool volumes, respectively. RS_CT_ for an endocardial surface voxel was calculated as the average RS_CT_ value of a patch of mesh faces directly connected with this voxel. RS_CT_ values were projected onto pixels in each VR video view (see Supplementary Material 2 for details about projection) to generate a ground truth map of endocardial function for each region from the perspective of each VR video. Then, each angular position was classified as abnormal (WMA present) if >35% of the endocardial surface in that view had impaired RS_CT_ (RS_CT_≥-0.20). Supplementary Material 2A explains how these thresholds were selected.

To do per-study classification in this project, we defined that a CT study is abnormal if it has more than one VR videos labeled as abnormal (N_ab_videos_ ≥ 2). Other thresholds (e.g., N_ab_videos_ ≥ 1 or 3) were also chosen and the corresponding results were shown in the Supplementary Material 3.

### DL framework design

**Figure 2:**
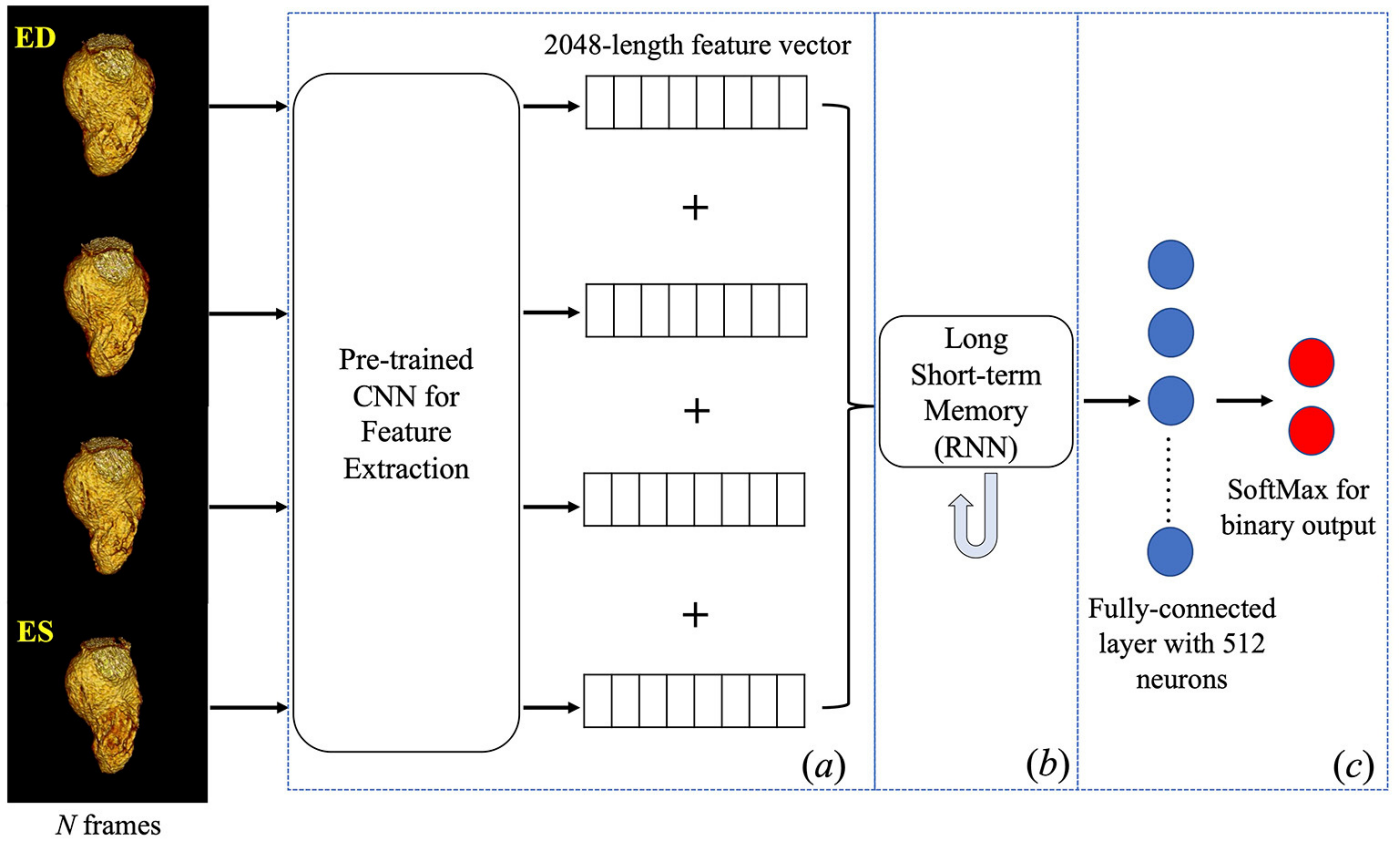
Deep learning framework. Four frames were input into a pre-trained inception-v3 individually to obtain a 2048-length feature vector for each frame. Four vectors were concatenated into a feature matrix which was then input to the next components in the framework. A Long Short-term Memory followed by fully connected layers was trained to predict a binary classification of the presence of WMA in the video. CNN, convolutional neural network; RNN, recurrent neural network.

The DL framework (see Figure 2) consists of three components, (*a*) a pre-trained 2D convolutional neural network (CNN) used to extract spatial features from each input frame of a VR video, (*b*) a recurrent neural network (RNN) designed to incorporate the temporal relationship between frames, and (*c*) a fully connected neural network designed to output the classification.

Given our focus on systolic function, four frames (ED, two systolic frames, and ES) were input to the DL architecture. This sampling was empirically found to maximize DL performance (32). Given the CT gantry rotation time, this also minimizes view sharing present in each image frame while providing a fuller picture of endocardial deformation. Each frame was resampled to 299×299 pixels to accommodate the input size of the pre-trained CNN.

Component (*a*) is a pre-trained CNN with the Inception architecture (Inception-v3) (33) and the weights obtained after training on the ImageNet (34) database. The reason to pick Inception-v3 architecture can be found in this reference (32). This component was used to extract features and create a 2048-length feature vector for each input image. Feature vectors from the four frames were then concatenated into a 2D feature matrix with size = (4, 2048).

Component (*b*) is a long short-term memory (35) RNN with 2048 nodes, tanh activation and sigmoid recurrent activation. This RNN analyzed the (4, 2048) feature matrix from component (*a*) to synthesize temporal information (RNN does this by passing the knowledge learned from the previous instance in a sequence to the learning process of the current instance in that sequence then to the next instance). The final component (*c*), the fully connected layer, logistically regressed the binary prediction of the presence of WMA in the video.

### Cross-validation and testing

In our DL framework, component (*a*) was pre-trained and directly used for feature extraction whereas components (*b*) and (*c*) were trained end-to-end as one network for WMA classification. Parameters were initialized randomly. The loss function was categorical cross-entropy.

The dataset was split randomly into 60% and 40% subsets. 60% (205 studies, 1230 videos) were used for 5-fold cross-validation, meaning in each fold of validation we had 164 studies (984 videos) to train the model and the rest 41 studies (246 videos) to validate the model. We report model performance across all folds. 40% (138 studies, 828 videos) were used only for testing.

### Experiment settings

We performed all DL experiments using TensorFlow on an 8-core Ubuntu workstation with 32 GB RAM and with a GeForce GTX 1080 Ti (NVIDIA Corporation, Santa Clara, CA, USA). The file size of each 4DCT study and VR video were recorded. Further, the time needed to run each step in the entire framework (including the image processing, VR video generation and DL prediction) on the new cases was recorded.

### Model performance and LVEF

The impact of systolic function, measured *via* LVEF on DL classification accuracy was evaluated in studies with LVEF < 40%, LVEF between 40-60%, LVEF >60%. We hypothesized that the accuracy of the model would be different for different LVEF intervals since because the “obviously abnormal” LV with low EF, and the “obviously normal” LV with high EF would be easier to classify. The consequence of a local WMA in hearts with LVEF between 40-60% might be a more subtle pattern and harder to detect. These subtle cases are also difficult for human observers.

### Comparison with expert visual assessment

While not the primary goal of the study we investigated the consistency of the DL classifications with the results from two human observers using traditional views. 100 CT studies were randomly selected from the testing cohort for independent analysis of WMA by two cardiovascular imaging experts with different levels of experiences: expert 1 with >20 years of experience (author A.K.) and expert 2 with >5 years of experience (author H.K.N.) The experts classified the wall motion in each AHA segment into 4 classes (normal, hypokinetic, akinetic and dyskinetic) by visualizing wall motion from standard 2D short- and long-axis imaging planes, in a blinded fashion. Because of the high variability in the inter-observer classifications of abnormal categories we: (1) combined the last three classes into a single “abnormal” class indicating WMA detection, and (2) we performed the comparison on a per-study basis. A CT study was classified as abnormal by the experts if it had more than one abnormal segment. The interobserver variability is reported in the result Section Model performance-comparison with expert assessment. It should be noted that our model was only trained on ground truth based on quantitative RS_CT_ values; the expert readings were performed as a measure of consistency with clinical performance.

### Statistical evaluation

Two-tailed categorical z-test was used to compare data proportions (e.g., proportions of abnormal videos) in two independent cohorts: a cross-validation cohort and a testing cohort. Statistical significance was set at *P* ≤ 0.05.

DL Model performance against the ground truth label was reported *via* confusion matrix and Cohen's kappa value. Both regional (per-video) and per-study comparison were performed. A CT study is defined as abnormal if it has more than one VR videos labeled as abnormal (N_ab_videos_ ≥ 2). As stated in Section Production of volume rendering video of LV blood-pool, every projection view of the VR video corresponded to a specific regional LV view. Therefore, we re-binned the per-video results into 6 LV views to test the accuracy of the DL model when looking at each region of the LV. We also calculated the DL per-study accuracy for patients with each clinical cardiac indication in the testing cohort and use pair-wise Chi-squared test to compare the accuracies between indications.

## Results

Of the 1230 views (from 205 CT studies) used for 5-fold cross-validation, 732 (from 122 studies, 59.5%) were male (age: 63 ± 15) and 498 (from 83 studies, 40.5%) were female (age: 62 ± 15). The LV blood pool had a median intensity of 516 HU (IQR: 433 to 604). 40.0% (492/1230) of the videos were labeled as abnormal based on RS_CT_ analysis, and 45.4% (93/205) of studies had WMA in ≥2 videos. 104 studies had LVEF > 60%, 54 studies had LVEF < 40% and the rest 47 (47/205 = 22.9%) studies had LVEF between 40-60%. For clinical cardiac indications, 85 studies have suspect CAD, 77 studies have the pre-PVI assessment, 31 studies have the pre-TAVR assessment, and 12 studies have the pre-VAD assessment.

Of the 828 views (from 138 CT studies) used for testing, 504 (from 84 studies, 60.9%) were male (age: 57 ± 16) and 324 (from 54 studies, 39.1%) were female (age: 63 ± 13). The LV blood pool had a median intensity of 520 HU (IQR: 442 to 629). 37.0% (306/828) of the videos were labeled as abnormal, and 45.0% (62/138) of studies had WMA in ≥2 videos. 72 studies had LVEF > 60%, 25 studies had LVEF < 40% and the rest 41 (41/138 = 28.7%) studies had LVEF between 40-60%. For clinical cardiac indications, 68 studies have suspect CAD, 49 studies have the pre-PVI assessment, 11 studies have the pre-TAVR assessment, and 10 studies have the pre-VAD assessment.

There were no significant differences (all *P*-values > 0.05) in data proportions between the cross-validation and testing cohorts in terms of the percentages of sex, abnormal videos, abnormal CT studies.

### Model performance—per-video and per-study classification

Per-video and per-study DL classification performance for WMA were excellent in both cross-fold validation and testing. Table 1 shows that the per-video classification for the *cross-validation* had high accuracy = 93.1%, sensitivity = 90.0% and specificity = 95.1%, Cohen's kappa κ = 0.86 with 95% CI as [0.83, 0.89]. Per-study classification also had excellent performance with accuracy = 93.7%, sensitivity = 93.5% and specificity = 93.8%, κ = 0.87[0.81, 0.94]. Table 1 also shows that the per-video classification for the *testing cohort* had high accuracy = 90.9%, sensitivity = 90.2% and specificity = 91.4%, κ = 0.81[0.77, 0.85]. We obtained per-study classification accuracy = 93.5%, sensitivity = 91.9% and specificity = 94.7%, κ = 0.87[0.78, 0.95] in the testing cohort.

**Table 1:** DL classification performance in cross-validation and testing.

		**Cross-validation**				**Testing**			
		**Per-video**		**Per-study (N**_**ab_videos**_ ≥ **2)**		**Per-video**		**Per-study (N**_**ab_videos**_ ≥ **2)**	
		**Ground truth**		**Ground truth**		**Ground truth**		**Ground truth**	
		**Abnormal**	**Normal**	**Abnormal**	**Normal**	**Abnormal**	**Normal**	**Abnormal**	**Normal**
DL	Abnormal	443	36	87	7	276	45	57	4
	Normal	49	702	6	105	30	477	5	72
		Sens	0.900	Sens	0.935	Sens	0.902	Sens	0.919
		Spec	0.951	Spec	0.938	Spec	0.914	Spec	0.947
		Acc	0.931	Acc	0.937	Acc	0.909	Acc	0.935
		κ	0.855	κ	0.872	κ	0.808	κ	0.868

*Two hundred five CT studies and 1230 Volume Rendered (VR) videos were used for 5-fold cross-validation. One hundred thirty-eight CT studies and 828 VR videos were in the testing. The four confusion matrices correspond to per-video classification (light gray) and per-study classification (dark gray) for cross-validation (left) and testing (right). N_ab_videos_ ≥2 (number of views classified as abnormal) was used to classify a study as abnormal. Sens, sensitivity; Spec, specificity; Acc, accuracy. Cohen's kappa κ is also reported*.

**Figure 3:**
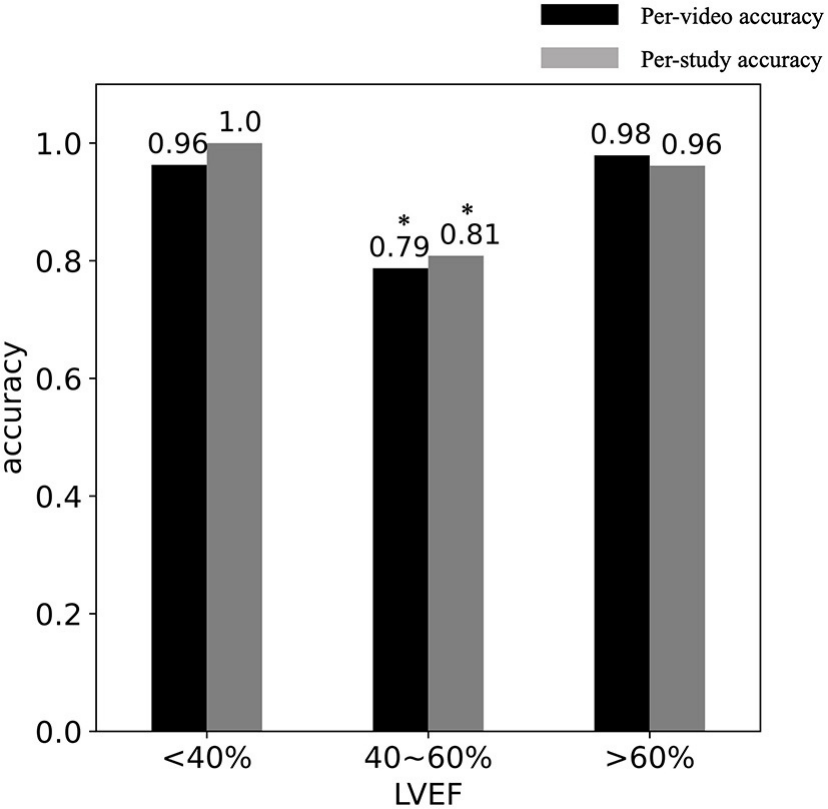
DL classification accuracy vs. LVEF. The per-video (black) and per-study (gray) accuracy are shown in studies with (LVEF < 40%), (40 < LVEF < 60%) and (LVEF > 60%). *Indicates the significant difference.

Figure 3 shows the relationship between DL classification accuracy and LVEF in the cross-validation. Table 2 shows that CT studies with LVEF between 40 and 60% in the cross-validation cohort were classified with per-video accuracy = 78.7%, sensitivity = 78.0% and specificity = 79.8%. In the testing cohort, per-video classification accuracy = 80.1%, sensitivity = 82.9% and specificity = 75.5% accuracy for this LVEF group remained relatively high but was lower (*P* < 0.05) than the accuracy for patients with LVEF < 40% and LVEF > 60% due to the more difficult nature of the classification task in this group with more “subtle” wall motion abnormalities.

**Table 2:** DL classification performance in CT studies with 40 < LVEF < 60%.

		**Cross-validation**				**Testing**			
		**Per-video**		**Per-study (N**_**ab_videos**_ **≥** **2)**		**Per-video**		**Per-study (N**_**ab_videos**_ **≥** **2)**	
		**Ground truth**		**Ground truth**		**Ground truth**		**Ground truth**	
		**Abnormal**	**Normal**	**Abnormal**	**Normal**	**Abnormal**	**Normal**	**Abnormal**	**Normal**
DL	Abnormal	131	23	33	5	126	23	32	3
	Normal	37	91	4	5	26	71	1	5
		Sens	0.780	Sens	0.892	Sens	0.829	Sens	0.970
		Spec	0.798	Spec	0.500	Spec	0.755	Spec	0.625
		Acc	0.787	Acc	0.809	Acc	0.801	Acc	0.902
		κ	0.567	κ	0.407	κ	0.581	κ	0.657

*Forty-seven CT studies with 40% < LVEF < 60% were in the cross-validation and 41 CT studies were in the testing. The light gray indicates per-video evaluation, dark gray indicates per-study evaluation*.

### Model performance—regional LV views

Table 3 shows that our DL model was accurate for detection of WMA in all 6 regional LV views both in cross-validation cohort (mean accuracy = 93.1% ± 0.03) and testing cohort (mean accuracy = 90.9% ± 0.06).

**Table 3:** Results re-binned into six regional LV views.

		**Per-video classification**							
		**Cross-validation**				**Testing**			
**Projection view**	**LV wall on the foreground**	**Sens**	**Spec**	**Acc**	κ	**Sens**	**Spec**	**Acc**	κ
0	Anterolateral	0.845	0.964	0.922	0.824	0.886	0.936	0.920	0.818
60	Inferolateral	0.938	0.952	0.946	0.888	0.909	0.915	0.913	0.805
120	Inferior	0.879	0.974	0.932	0.860	0.917	0.910	0.913	0.824
180	Inferoseptal	0.882	0.946	0.917	0.832	0.847	0.861	0.855	0.705
240	Anteroseptal	0.963	0.944	0.951	0.899	0.927	0.952	0.942	0.879
300	Anterior	0.893	0.931	0.917	0.822	0.932	0.904	0.913	0.807

*This table shows the per-video classification of our DL model when detecting WMA from each regional view of LV. See the definition of regional LV views in Section Production of volume rendering video of LV blood-pool. Sens, sensitivity; Spec, specificity; Acc, accuracy*.

### Model performance—different clinical cardiac indications

We calculated the DL per-study classification accuracy equal to 91.2% for CT studies with suspect CAD (*n* = 68 in the testing cohort), 93.9% for studies with pre-PVI assessment (*n* = 49), 100% for patients with pre-TAVR assessment (*n* = 11), 100% for studies with pre-LVAD assessment (*n* = 10). Using Chi-squared test pairwise, there was no significant difference of DL performance between indications (all *P*-values > 0.5).

### Model performance—comparison with expert assessment

First, we report the interobserver variability of two experts. The Cohen's kappa for the agreement between observers on per-AHA-segment basis was 0.81[0.79, 0.83] and on the per-CT-study basis was 0.88[0.83, 0.93]. For those segments labeled as abnormal by both experts, the Kappa for the two experts to further classify an abnormal segment into hypokinetic, akinetic and dyskinetic dramatically dropped to 0.34.

Second, we show in the Table 4 that per-study comparison between DL prediction and expert visual assessment on 100 CT studies in the testing cohort led to Cohen's Kappa κ = 0.81[0.70,0.93] for expert 1 and κ = 0.73[0.59,0.87] for expert 2.

**Table 4:** Comparison between DL and expert visual assessment.

		**Expert visual assessment**			
		**Expert 1**		**Expert 2**	
		**Abnormal**	**Normal**	**Abnormal**	**Normal**
DL	Abnormal	37	5	33	9
	Normal	4	54	4	54
		κ	0.815	κ	0.729

*Per-study comparison were run on 100 CT studies randomly selected from the testing cohort. The light gray indicates per-video evaluation, dark gray indicates per-study evaluation*.

### Data-size reduction

The average size of the CT study across one cardiac cycle was 1.52 ± 0.67 Gigabytes. One VR video was 341 ± 70 Kilobytes, resulting in 2.00 ± 0.40 Megabytes for 6 videos per study. VR videos led to a data size that is ~800 times smaller than the conventional 4DCT study.

### Run time

Regarding image processing, the image rotation took 14.1 ± 1.2 seconds to manually identify the landmarks and then took 38.0 ± 16.2 seconds to automatically rotate the image using the direction vectors derived from landmarks. The DL automatic removal of unnecessary structures took 141.0 ± 20.3 seconds per 4DCT study. If needed, manual pacing lead artifacts removal took around 5–10 mins per 4DCT study depending on the severity of artifacts. Regarding automatic VR video generation, it took 32.1 ± 7.0 seconds (to create 6 VR videos from the processed CT images). Regarding DL prediction of WMA presence in one CT study, it took 0.7 ± 0.1 seconds to extract image features from frames of the video and took ~0.1 seconds to predict binary classification for all 6 VR videos in the study. To summarize, the entire framework requires approximately 4 minutes to evaluate a new study if no manual artifacts removal is needed.

## Discussions

In this study, we developed and evaluated a DL framework that detects the presence of WMA in dynamic 4D volume rendering (VR videos) depicting the motion of the LV endocardial boundary. VR videos enabled a highly compressed (in terms of memory usage) representation of large regional fields of view with preserved high spatial-resolution features in clinical 4DCT data. Our framework analyzed four frames spanning systole extracted from the VR video and achieved high per-video (regional LV view) and per-study accuracy, sensitivity and specificity (≥0.90) and concordance (κ≥ 0.8) both in cross-validation and testing.

### Benefits of the volume visualization approach

Assessment of regional WMA with CT is usually performed on 2D imaging planes reformatted from the 3D volume. However, 2D approaches often confuse the longitudinal bulk displacement of tissue into and out of the short-axis plane with true myocardial contraction. Various 3D analytical approaches (9, 20, 28) to quantify 3D motion using image registration and deformable LV models have been developed; our novel use of regional VR videos as input to DL networks has several benefits when compared to these traditional methods. First, VR videos contain 3D endocardial surface motion features which are visually apparent. This enables simultaneous observation of the complex 3D motion of a large region of the LV in a single VR video instead of requiring synthesis of multiple 2D slices. Second, our framework is extremely memory efficient with reduced data size while preserving key anatomical and motion information; a set of 6 VR videos is ~800 times smaller in data size than the original 4DCT data. The use of VR videos also allows our DL experiments to run on the current graphic processing unit (GPU), whereas the original 4DCT data is too large to be imported into the GPU. Third, our framework is simple as it does not require complex and time-consuming computations such as point registration or motion field estimation included in analytical approaches. The efficiency of our technique will enable retrospective analysis of large numbers of functional cardiac CT studies; this cannot be said for traditional 3D tracking methods which require significant resources and time for segmentation and analysis.

### Model performance for each LV view

We re-binned the per-video results into 6 projection views corresponding to 6 regional LV views and showed that our DL model is accurate to detect WMA from specific regions of the LV. The results shown in Table 3 indicate that all results for classification can be labeled with a particular LV region. For example, to evaluate the wall motion on the inferior wall of a CT study, the classification from the VR video with the corresponding projection view θ(=120) would be used.

### Comparison with experts and its limitations

To evaluate the consistency of our model with standard clinical evaluation, we compared DL results with two cardiovascular imaging experts and showed high per-study classification correspondence. This comparison study has its limitations. First, we did not perform a per-AHA-segment comparison. Expert visual assessment was subjective (by definition) and had greater inter-observer variability on per-AHA-segment basis than the per-study basis the variability (Kappa increased from 0.81 for per-segment to 0.88 for per-study). Second, the interobserver agreement for experts to further classify an abnormal motion as hypokinetic, akinetic or dyskinetic was also too poor (Kappa = 0.34) to use expert visual labels for three severities as the ground truth; therefore, we used one “abnormal” class instead of three levels of severity of WMA. Third, experts could only visualize the wall motion from 2D imaging planes while our DL model evaluated the 3D wall motion from VR videos. A future study using a larger number of observers, and a larger number of cases could be performed in which trends could be observed; however, it is clear that variability in subjective calls for degree of WMA will likely persist in the expert readers.

### Using RS_CT_ for ground truth labeling

Direct visualization of wall motion abnormalities in volume rendered movies from 4DCT is a truly original application; hence, as can be expected there are no current clinical standards/guidelines for visual detection of WMA from volume rendered movies. In fact, we believe our paper is the first to introduce this method of evaluating myocardial function in a formal pipeline. In our recent experience, visual detection of patches of endocardial “stasis” in these 3D movies highly correlates with traditional markers of WMA such as wall thickening, circumferential shortening and longitudinal shortening. However, specific guidance on how to clinically interpret VR movies is not yet available. We expect human interpretation to depend on both experience and training. Thus, we used quantitative regional myocardial shortening (RS_CT_) derived from segmentation and 3D tracking to delineate regions of endocardial WMA. RS_CT_ has been previously shown to be a robust method for quantifying regional LV function (8, 12, 28, 29).

### Limitations and future directions

First, our current DL pipeline has several manual image processing such as manual rotation of the image and manual removal of lead artifacts. These steps lengthen the time required to run the entire pipeline (see Section Run time) and limit the clinical utility. One important future direction of our technique is to integrate the DL-driven automatic image processing to get a fully automatic pipeline. Chen et al. (26) have proposed a DL technique to define the short-axis planes from CT images so that the LV axis can be subsequently derived for correct image orientation. Zhang and Yu (36) and Ghani and Karl (37) have proposed DL techniques to remove the lead artifacts.

Second, our work only focuses on the systolic function and only takes 4 systolic frames from the VR video as the model input. The future direction is to input diastolic frames into the model to enable the evaluation of diastolic function and to use a 4D spatial-temporal convolutional neural network (38) to directly process the video without requiring explicit selection of temporal frames.

Third, we currently perform binary classification of the presence of WMA in the video. The DL model integrates all information from all the AHA segments that can be seen in the video and only evaluates the extent of pixels with WMA (i.e., whether it's larger than 35% of the total pixels). The DL evaluation is independent of the position of WMA; thus, we do not identify which of the AHA segments contribute to the WMA just based on the DL binary classification. Future research is needed to “focus” the DL model's evaluation on specific AHA segments using such as local attention (39) and evaluate whether the approach can delineate the location and extent of WMA in terms of AHA segments. Further, by using a larger dataset with a balanced distribution of all four severities of WMA, we aim to train the model to estimate the severity of the WMA in the future.

Fourth, tuning the inceptionV3 (the CNN) weights to extract features most relevant to detection of WMA is expected to further increase performance as it would further optimize how the images are analyzed. However, given our limited training data, we chose not to train weights of the inception network and the high performance we observed seems to have supported this choice.

In conclusion, we developed a framework that combines the video of the volume rendered LV endocardial blood pool with deep learning classification to detect WMA and observed high per-region (per-video) and per-study accuracy. This approach has promising clinical utility to screen for cases with WMA simply and accurately from highly compressed data.

## Data availability statement

The original contributions presented in the study are included in the article/supplementary material, further inquiries can be directed to the corresponding author/s.

## Ethics statement

The studies involving human participants were reviewed and approved by University of California, San Diego, Human Research Protections Program, IRB_approval_#191797. Written informed consent for participation was not required for this study in accordance with the national legislation and the institutional requirements.

## Author contributions

ZC, FC, and EM designed the overall study and performed the final analysis. ZC developed, trained, and validated the deep learning network, collected all the retrospective cardiac 4DCT studies, performed data curation, and drafted the whole manuscript. GC, AM, and ZC designed the pipeline to measure RS_CT_. AK and HN provided the expert visual assessment on 100 CT studies. All authors participated in the analysis, interpretation of data, revising the manuscript critically, and final approval of the submitted manuscript.

## Funding

FC was supported by NHLBI HL145817, HL143113, and HL144678. EM and ZC were supported by HL144678 and HL153250. AM was supported by AHA 20PRE35210261.

## Conflict of interest

Author EM has founder shares in Clearpoint Neuro Inc. The remaining authors declare that the research was conducted in the absence of any commercial or financial relationships that could be construed as a potential conflict of interest.

## Publisher's note

All claims expressed in this article are solely those of the authors and do not necessarily represent those of their affiliated organizations, or those of the publisher, the editors and the reviewers. Any product that may be evaluated in this article, or claim that may be made by its manufacturer, is not guaranteed or endorsed by the publisher.
