# Supplementary material for: Detection of left ventricular wall motion abnormalities from volume rendering of 4DCT cardiac angiograms using deep learning
Source: Front Cardiovasc Med. 2022 Jul 28;9:919751. doi: 10.3389/fcvm.2022.919751 (PMC9366190; doi:10.3389/fcvm.2022.919751)
Supplement: Supplementary file 1 [file Data_Sheet_1.pdf]

SUPPLEMENTAL MATERIALS

1. Volume Rendering in MATLAB

A. Pre-set parameters for Volume Rendering

Built-in volume rendering function in MATLAB called "volshow" was used to automatically generate VR from 3D CT volume. Since in the preprocessing every CT volume was rotated to have a uniform orientation, a same set of camera-related parameters could be used across the entire dataset:"CameraPosition" was [6,0,1], "CameraUpVector" was [0,0,1], "CameraViewAngle" was 15°. CT image was normalized based on the study-specific window level and window width. See section "automated volume rendering video generation" in the main text for how to set these window level and window width. The built-in colormap ("hot") and a linear alphamap was applied to the normalized CT image, assigning colors and opacities to each voxel according to its intensity. The background color was set to be black, and the lighting effect was turned on.

B. Production of Six VR Videos for Each Study

Each VR video shows the projection of the 3D CT volume at one specific view angle θ. To evaluate all AHA segments, 6 VR videos, with six different views $\theta_{60\times n,\ n\in[0,1,2,3,4,5]}$ corresponding to 60-degree clockwise rotations around the LV long axis, were generated for each study. The rotation of the camera was done automatically by applying a rotation matrix to the parameter "CameraPosition" for each video. The rotation was around the LV long axis which is the z-axis of the image, so the "CameraPosition" for a video with view angle θ can be calculated as:

$$\begin{bmatrix} px \\ py \\ pz \end{bmatrix} = \begin{bmatrix} cos(\theta) & -sin(\theta) & 0 \\ sin(\theta) & cos(\theta) & 0 \\ 0 & 0 & 1 \end{bmatrix} \times \begin{bmatrix} 6 \\ 0 \\ 1 \end{bmatrix}$$

where [6 0 1] is the pre-set "CameraPosition" and [px py pz] is the derived "CameraPosition" for each view angle. All other rendering parameters were kept constant for every video.

2. Video Classification for the Presence of Wall Motion Abnormality

This section explains how we classified the WMA presence in a VR video with view angle θ based on the per-voxel RS_{CT} map for the endocardium.

The pipeline follows the steps below:

Step 1. Binarize the per-voxel RS_{CT} map using a threshold RS_{CT}*.

Step 2. Use the MATLAB built-in function "labelvolshow" to get the rendering image $\mathcal{R}_{RS}$ of the binary RS_{CT} map with the same view angle θ of the VR video (see an example of labeled rendering $\mathcal{R}_{RS}$ in **Figure 1 step c**). "Labelvolshow" is a function to display the rendering of labeled volumetric data. All camera-related rendering parameters were kept the same as those for the VR video. As a result, $\mathcal{R}_{RS}$ displays the same endocardial surface as the VR video does.

Step 3. Count the number of abnormal pixels in $\mathcal{R}_{RS}$ and calculate its percentage = $\frac{n_{abnormal\,pixels}}{n_{abnormal\,pixels}+n_{normalpixels}} \times 100\%$. A VR video is labeled as abnormal if >35% of the pixels in the $\mathcal{R}_{RS}$ (equivalently, >35% of the endocardial surface of LV) are abnormal.

<u>In conclusion, a VR video was classified as abnormal (presence of WMA) if >35% of the endocardial surface of the LV had RS_{CT} ≥RS_{CT}*. Here we set RS_{CT}* = -0.20.</u>

2.A Threshold Value Choices

A VR video is classified as abnormal if >35%. Here > 35% was set based on the following derivation: since each projected view showed 3 AHA walls, if one AHA wall has WMA then approximately one-third (≈35%) of the projected CT would have abnormal RS_{CT}.

The threshold $RS_{CT}* \geq -0.20$ was set based on the previous research by Colvert et al[29]. They showed the average RS_{CT} for a cohort of 23 healthy controls is equal to -0.32 ± 0.06. Our threshold number $RS_{CT}*$ to detect abnormal regions (WMA) was two standard deviations above the mean in normal cases = -0.20.

3. Per-study Classification with Different Threshold N_{ab_videos}

Supplemental Table 1: per-study classification when a study is defined as abnormal with at least one VR video labeled as abnormal ($N_{ab\_videos} \geq 1$)

		Cross-Validation		Testing	
		Ground Truth		Ground Truth	
		Abnormal	Normal	Abnormal	Normal
DL	Abnormal	92	9	62	14
	Normal	4	100	4	58
		Sens	0.958	Sens	0.939
		Spec	0.917	Spec	0.806
		Acc	0.937	Acc	0.870
		κ	0.873	κ	0.740

Supplemental Table 2: per-study classification when a study is defined as abnormal with more than two VR video labeled as abnormal ($N_{ab\_videos} \geq 3$)

		Cross-Validation		Testing	
		Ground Truth		Ground Truth	
		Abnormal	Normal	Abnormal	Normal
DL	Abnormal	81	5	52	1
	Normal	6	113	5	80

	Sens	0.931	Sens	0.912
	Spec	0.958	Spec	0.988
	Acc	0.946	Acc	0.957
	κ	0.890	κ	0.909
